# Hospital Claims and the Insurance Surplus

**Published:** 1921-05-21

**Authors:** 


*To the Editor of* THE HOSPITAL.

### Hospital Claims and the Insurance Surplus.

*To the Editor of* THE HOSPITAL.

SIR,—I observe that the National Deposit Friendly Society is about to allocate one-third of its disposable surplus, under the National Insurance Act, accumulated during a period of five years, to hospitals and nursing organisations. This amount will total £127,000. The management of all hospitals will welcome the excellent example set by this society. I think it may be said that public opinion has now been educated to the recommendation recently made by Lord Cave's Committee that, in future, insurance societies should be expected to pay a large share of the cost of the medical treatment of their insured members, but I think the views of hospitals go a little further than this, and it is generally felt by them that a lump sum should be paid them down at once to recoup them for the services they have rendered to members of approved societies in the past—that is to say, during the five years in which the large surplus of £7,000,000 was accumulated by the various societies. Certainly they should bear the cost of expenses in the future; but with equal justice should they be asked to pay off some of the arrears of debt they owe the voluntary hospitals.—Yours obediently,

NORTHAMPTON,
Chairman of the Great Northern Hospital.

10 Berkeley Street, W. 1.

